# CLIP-related methodologies and their application to retrovirology

**DOI:** 10.1186/s12977-018-0417-2

**Published:** 2018-05-02

**Authors:** Paul D. Bieniasz, Sebla B. Kutluay

**Affiliations:** 1 Howard Hughes Medical Institute and Laboratory of Retrovirology, The Rockefeller University, New York, NY 10065 USA; 2 Department of Molecular Microbiology, Washington University School of Medicine, Saint Louis, MO 63110 USA

## Abstract

Virtually every step of HIV-1 replication and numerous cellular antiviral defense mechanisms are regulated by the binding of a viral or cellular RNA-binding protein (RBP) to distinct sequence or structural elements on HIV-1 RNAs. Until recently, these protein–RNA interactions were studied largely by in vitro binding assays complemented with genetics approaches. However, these methods are highly limited in the identification of the relevant targets of RBPs in physiologically relevant settings. Development of crosslinking-immunoprecipitation sequencing (CLIP) methodology has revolutionized the analysis of protein–nucleic acid complexes. CLIP combines immunoprecipitation of covalently crosslinked protein–RNA complexes with high-throughput sequencing, providing a global account of RNA sequences bound by a RBP of interest in cells (or virions) at near-nucleotide resolution. Numerous variants of the CLIP protocol have recently been developed, some with major improvements over the original. Herein, we briefly review these methodologies and give examples of how CLIP has been successfully applied to retrovirology research.

## Background

Following the integration of proviral DNA into the host cell chromosome, genesis of new HIV-1 particles is initiated by the host RNA Polymerase II-mediated synthesis of a single poly-cistronic viral RNA species [1]. This transcript undergoes varying levels of alternative splicing generating over 40 different RNA species, an event orchestrated by the host cellular splicing machinery and cis-acting elements on viral RNAs [1, 2]. Like cellular mRNAs, all viral RNAs contain 5′ 7-methylguanosine (m7G) caps and 3′ polyA tails [1, 3]. While fully spliced viral RNAs can exit the nucleus via canonical nuclear export pathways, the partially spliced and unspliced viral RNAs depend on the viral Rev and cellular Crm1 proteins for nuclear export [4]. All viral mRNAs are subsequently translated in the cytosol, but the unspliced full-length viral RNAs also serve as the viral genome and are packaged into virions by the viral major structural protein Gag. Following their release from the plasma membrane, particles undergo a maturation step triggered by the viral protease enzyme. During this process, Gag and Gag-Pol proteins are cleaved into their constituent domains, the CA domain of Gag forms a conical lattice and the viral RNA genome condenses with the cleaved NC domain of Gag and viral enzymes inside this conical core [5, 6]. Thus, virtually every step in HIV-1 replication depends on a complex and changing set of interactions between viral RNAs and the multitude of trans-acting viral and cellular RNA-binding proteins. Historically, the interactions between these proteins and their RNA targets have largely been mapped by genetic studies, complemented by limited in vitro approaches. Comprehensive analysis of these interactions in physiologically relevant settings was effectively impossible prior to the recent development of cutting-edge next-generation sequencing-based methodologies. These methods, collectively referred to as CLIP (crosslinking-immunoprecipitation coupled with next-generation sequencing), allow the global identification of RNA targets of RNA-binding proteins (RBPs) in physiological settings in unprecedented detail. In this review, we provide a detailed outline of the existing CLIP methodologies, discuss their advantages and shortcomings (based partly on our own experience) and give examples of how CLIP has been successfully applied to retrovirology research.

## Principles of CLIP and variant methodologies

**Fig. 1:**
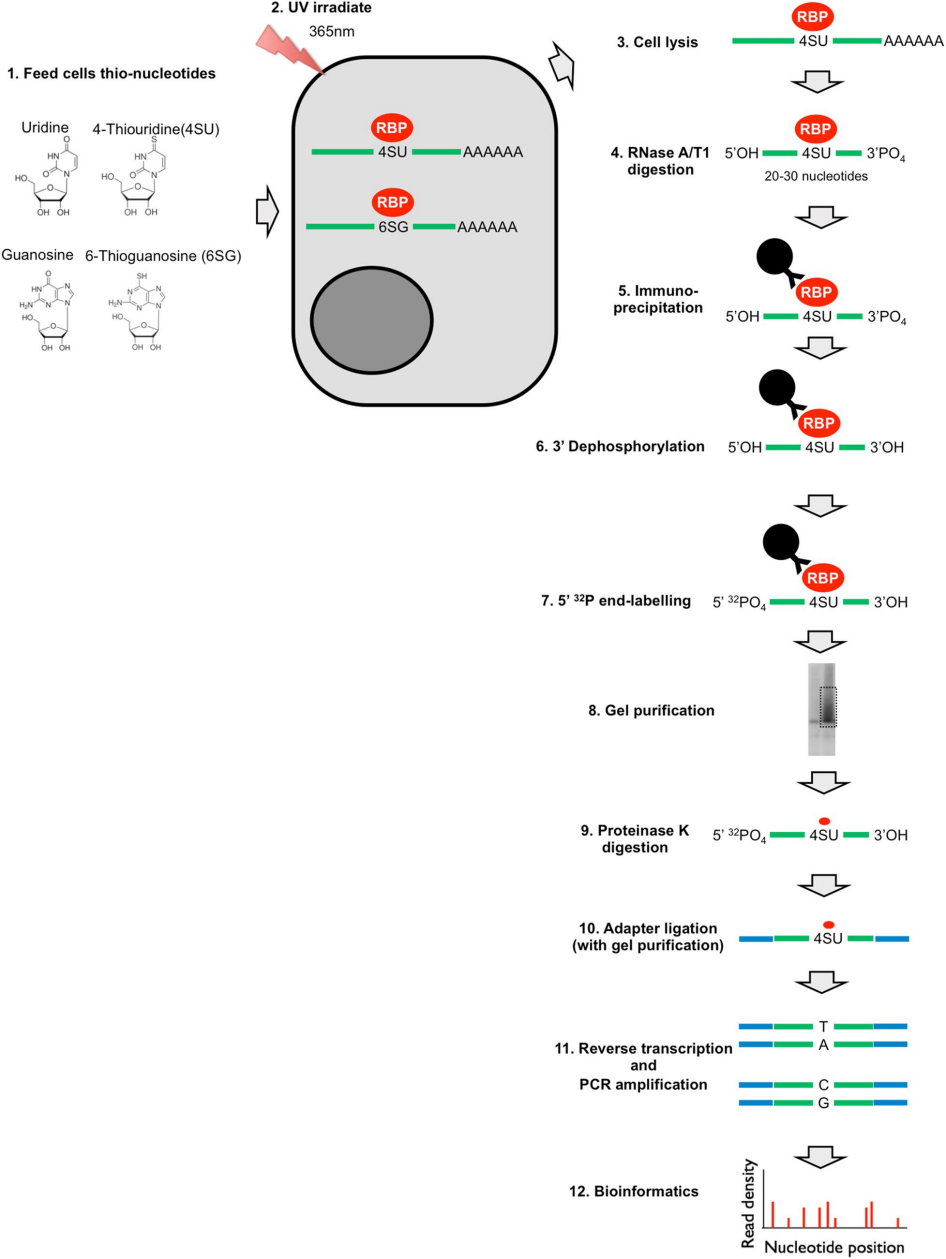
Outline of CLIP

In simple terms, CLIP is a powerful methodology with which one can identify the RNA targets of RNA-binding proteins in physiological settings, ranging from live cells to virus particles and even animal tissues. The inception of the original CLIP protocol [7, 8] and its subsequent coupling to next-generation sequencing [9] has revolutionized the study of protein–RNA interactions. Since then, several other versions of CLIP have been developed. The salient steps of the existing CLIP methodologies are (Fig. 1): (1) protein–RNA complexes are covalently crosslinked in live cells/tissues/virions; (2) Cells/tissues/virions are lysed and treated with limited amounts of RNases leaving small fragments of RNA molecules (~ 20 to 50 nucleotides) protected by the protein of interest; (3) Protein–RNA complexes are immunoprecipitated, and non-specific RNAs and proteins are removed by stringent washes. Because the protein–RNA complexes are covalently crosslinked, these stringent conditions, in principle, do not affect purification of target protein–RNA adducts. (4) The purified protein–RNA complexes are radioactively labeled and separated by SDS-PAGE. (5) Bound RNA is isolated either directly from SDS-PAGE gels or from nitrocellulose membranes following transfer by Proteinase K treatment. (6) Eluted RNA is ligated to adapters, reverse transcribed, the resulting cDNA is PCR amplified and subjected to sequencing. (7) Sequencing reads are processed and mapped to reference genomes. Depending on the method used, the resulting library contains nucleotide substitutions or deletions at the site of crosslinking, which allows mapping of the site of protein–RNA interactions at near-nucleotide resolution. Subsequent analyses include determination of the significantly enriched binding sites, identification of the binding motifs within them as well as other custom analyses. In the remainder of this section we will review the currently existing CLIP methods and give an overview of the widely used CLIP data analysis tools and pipelines.

### HITS-CLIP

Historically, protein–RNA interactions were studied largely using in vitro binding assays with pure proteins and RNAs. Alternatively, GST-pulldown and immunoprecipitation-based assays were conducted on cell lysates followed by downstream quantitative analysis of RNA by Q-RT-PCR or microarrays. A major drawback of these cell lysate-based approaches was their limited ability to identify direct interactions between a RBP and its target RNA molecules. Their limited power was due at least in part to the presence of contaminating protein and RNA molecules in the isolated RBP-RNA complexes. Development of the original CLIP protocol [7, 8], in which the protein–RNA complexes were UV-crosslinked in vivo and immunoprecipitated under stringent conditions to remove the contaminating proteins and RNA molecules marked the first advancement over these traditional methods. While the initial CLIP methodology relied on cloning and subsequent sequencing of the RNA targets, the coupling of CLIP to high-throughput sequencing, HITS-CLIP, allowed global transcriptome-wide analysis of RBP-RNA crosslinks [9]. HITS-CLIP relies on UV crosslinking of protein–RNA complexes at UV254 nm. As such, HITS-CLIP can be applied to animal tissues due to its high level of penetration. Following crosslinking and immunoprecipitation of protein–RNA complexes, ligation to the radioactively labeled 5′ adapter is performed while the protein–RNA adducts are attached to beads. This allows the removal of unligated 5′ adapter by further rounds of bead washing, which substantially reduces the appearance of adapter–adapter ligation products following downstream processing. The isolated protein–RNA adducts are separated by SDS-PAGE and transferred to nitrocellulose membranes. As naked RNA molecules are not retained on the nitrocellulose membranes, protein–RNA complexes are purified further during this step. Transfer to nitrocellulose membranes has been utilized in other CLIP approaches and in our experience confers a major advantage over the originally described PAR-CLIP approach described below. Protein-crosslinked RNA is further purified from nitrocellulose membranes by proteinase K treatment, ligated to the 3′ adapters and PCR-amplified prior to sequencing. Detailed bioinformatics analyses of HITS-CLIP datasets revealed that reverse transcriptase (RT) introduces deletions at the site of crosslinking [10], albeit at a fairly low frequency, allowing HITS-CLIP to reach to near nucleotide-resolution identification of binding sites.

### PAR-CLIP

A major advantage of PAR-CLIP [11] over HITS-CLIP is the use of ribonucleoside analogs, including 4-thiouridine (4SU) and 6-thioguanosine (6SG), that significantly enhance the efficiency of protein–RNA crosslinking. In PAR-CLIP experiments, cells are typically grown in the presence of ribonucleoside analogs for up to 16 h and UV-crosslinked at a longer wavelength (365 nm). As such, in contrast to HITS-CLIP, application of PAR-CLIP is largely limited to cell culture systems (an exception being *C. elegans* which can be grown in 4SU containing media and efficiently UV-crosslinked due to its transparency [12]). Although the original PAR-CLIP description utilized an inducible tagged RNA-binding protein [11], we and many other groups have successfully adapted PAR-CLIP to study endogenous proteins, including HIV-1 NC and IN [13, 14], Argonaute [15–18], as well as other proteins involved in RNA biogenesis and metabolism [19–24]. A potential disadvantage of the PAR-CLIP protocol is the cellular toxicity that may be induced by 4SU treatment depending on the cell type, the dose and incubation time [25]. Thus, optimal conditions that allow efficient protein–RNA crosslinking without major toxicity should be determined on a case-by-case basis. Nevertheless, PAR-CLIP allows accurate nucleotide resolution mapping of target RNA sites due to mutations introduced by RT (T-to-C for 4SU and G-to-A for 6SG) precisely at the site of crosslinking during cDNA synthesis. While allowing nucleotide resolution mapping, use of ribonucleoside analogs may inadvertently enrich RNA elements with distinct nucleotide composition or alter RNA structure [26], which may subsequently affect protein binding. Careful validation of PAR-CLIP experiments with different ribonucleoside analogs and RNases should, in principle, address these potential problems.

### iCLIP

Identification of the precise crosslinking site in the HITS-CLIP and PAR-CLIP approaches relies respectively on deletions and substitutions introduced by RT during cDNA synthesis. However, read-through at crosslinking sites appears to be a relatively rare event as compared to truncations that occur as a result of RT stalling at these sites [27, 28]. Thus, a major shortcoming of HITS-CLIP and PAR-CLIP approaches is the loss of a large fraction (estimated to be > 80%) of the starting material due to the inability to recover truncated reverse transcription products. iCLIP [29] has been designed to address this problem by ligation of a 3′ adapter while protein–RNA complexes are still on beads followed by introduction of a two-part cleavable adaptor into cDNA during reverse transcription. The resulting cDNA is circularized and subsequently linearized with a restriction enzyme, which allows the recovery of a larger fraction of truncated cDNAs. In addition, as circularization is done at high temperatures, structured cDNA molecules are recovered at a much higher efficiency. As a result of this enrichment, iCLIP can yield higher complexity libraries and has been proposed to perform better than previous approaches in identification of the precise site of crosslinking [28–30]. Application of iCLIP on a large scale by the ENCODE consortium indicated that the success rate in generating libraries was low for many RBPs, which was ascribed to the low efficiency of the circularization step [31]. However, several studies that utilized iCLIP have generated libraries with sufficiently high complexity and sensitivity, and these parameters were not carefully assessed by the ENCODE consortium. The remainder of the iCLIP protocol is similar to HITS-CLIP and PAR-CLIP approaches. BrdU-CLIP [32] and FAST-iCLIP [33] are iterations of the iCLIP protocol, which provide alternative cDNA and RNA purification methods, respectively. For example, by exchanging the 3′ ddC blocker from the standard iCLIP 3′ adaptor with a 3′ biotin moiety and subsequent purification of ligation products on streptavidin beads, FAST-iCLIP is reported to reduce the time required to perform iCLIP by 50%.

### eCLIP

The eCLIP protocol [31] proposes to address some of the shortcomings of previous CLIP approaches by including two separate adapter ligation steps (i.e. in the HITS-CLIP and PAR-CLIP protocols). In eCLIP, the immunoprecipitated RNA is first ligated to an indexed 3′ RNA adapter while complexes are still on the immunoprecipitation beads, and to a 3′ single-stranded (ss) DNA adapter after reverse transcription. As reverse transcription frequently terminates at the RBP-RNA crosslinking site, the ligation of the 3′ ssDNA adapter to the terminated cDNA fragments allows higher recovery rates of the starting material and helps in identification of the binding sites as in iCLIP. In addition, as the first 3′ RNA adapter already contains the indeces, samples can be combined at an earlier stage than in other protocols saving processing time. While adapter ligations conducted on beads has been inefficient in our hands (see below), the authors suggest that increased T4 RNA ligase concentration and the addition of high concentrations of polyethylene glycol (PEG8000) and DMSO in ligation reactions enable ligation efficiencies of up to > 90% [31]. In addition, RNA radiolabeling and autoradiographic visualization steps can be omitted allowing even faster library preparation times. However, these steps in our experience are highly important to purify the target protein–RNA complexes away from other proteins and RNA molecules that have non-specifically immunoprecipitated. Thus, the specificity of eCLIP libraries should be carefully evaluated, as also reviewed by a recent study [34]. Finally, inclusion of a size-matched input control (SMInput) in eCLIP enables efficient background normalization and controls for any inherent biases in library generation. The remainder of the eCLIP protocol shares many of the same steps as other CLIP approaches, in particular iCLIP.

### irCLIP

Similar to eCLIP, irCLIP has been developed to overcome some of the shortcomings of previous CLIP methodologies by simplifying the library generation steps, increasing the yield and complexity of the CLIP library, and allowing faster processing times. One of the major differences of this approach is the utilization of a 3′ adapter conjugated to an infrared fluorescent dye [35], which provides a more sensitive and faster way of tracking the target RNA molecules compared to radioactive labeling. Similar to FAST-iCLIP, the adapter ligated RNA library is purified by streptavidin beads. CLIP has an inherent bias against identification of protein binding events on structured RNA elements due to stalling of RT at these sites. Although not proven, irCLIP may mitigate this problem by utilizing thermostable enzymes for circularization and reverse transcription steps to take place at 60 °C, which helps to resolve potential RNA secondary structures [35]. Other aspects of the irCLIP protocol, such as on-bead nuclease digestions and Proteinase K digestion in SDS have previously been utilized within the context of PAR-CLIP experiments [11, 14]. As in iCLIP and eCLIP, the irCLIP procedure achieves single-nucleotide resolution by recovery of truncated cDNAs after the reverse transcription stage.

### Customizing CLIP

The major shortcomings of all of the above CLIP approaches include technically challenging and labor-intensive protocols, and loss of the starting material at several inefficient steps in the procedure. This problem is further exacerbated if the initial protein–RNA complexes are not abundant due to low levels of expression in cells (virions), low crosslinking or immunoprecipitation efficiencies. These problems can often lead to a final library with insufficient complexity and enrichment of environmental contaminating sequences. When we adapted the CLIP protocol to study HIV-1 Gag-RNA interactions [36], we took advantage of both HITS-CLIP and PAR-CLIP protocols as detailed in [14]. In our experience, 4-SU-mediated crosslinking yielded more abundant Gag-, MA- and NC-RNA complexes, that was critical for generating libraries with sufficient sequence diversity for successful sequencing. While the original PAR-CLIP protocol relied on electroelution of protein–RNA complexes from SDS-PAGE gels, we opted for transfer of protein–RNA complexes to nitrocellulose membranes following SDS-PAGE (as in HITS-CLIP). As naked RNA oligonucleotides are not immobilized on nitrocellulose membranes, this step provides an added level of protein–RNA complex purification. While the HITS-CLIP and many other protocols call for ligation of adapters while the protein–RNA complexes are on beads, the PAR-CLIP library generation protocol in solution was significantly more efficient in our hands with 3′ and 5′ adapter ligations routinely working at > 90 and 50% efficiency. Although seemingly more cumbersome, sequential ligation of adapters provides more control over monitoring the ligation efficiency and substantially decreases contaminating adapter–adapter ligation products. Additionally, we have utilized barcoded and degenerate sequence containing adapters, which enabled us to combine multiple samples (typically up to eight) and distinguish between independent ligation versus PCR overamplification events, respectively. Finally, due to some of the potential inherent biases of the PAR-CLIP approach discussed above, we typically validate our findings using different ribonucleosides (4SU vs. 6SG) and RNases (RNase A vs. RNase T1).

### CLIP data analyses

CLIP data analyses can be summarized in four major steps: (1) pre-processing of sequencing reads. (2) mapping of reads to reference genomes, (3) subjecting mapped reads to cluster finding algorithms to define binding sites, (4) analysis of binding sites for enrichment of certain features including where within a gene body the binding site is located, presence of distinct motifs or nucleotide composition. Recently a few pipelines that can perform the majority of these steps have been developed and include the PARCLIPsuite [37], CLIPZ [38], CIMS [39] and CLIP-seq tools [40]. Below, we will go through some of the publicly available and most frequently used standalone tools that can be utilized for analyses of CLIP data sets. For a more detailed review of these tools and algorithms we refer the readers to detailed recent reviews [41–44]. Implementing many of these analysis pipelines requires some level of coding knowledge and familiarity with shell scripting.

1. Pre-processing of sequencing reads: The resulting CLIP libraries in all of the above protocols will contain some form of 3′ and 5′ adaptors. In the majority of cases, these adaptors contain barcodes and degenerate sequences (N_3–10_), which allow multiplexing and differentiating between independent ligation versus PCR overamplification events, respectively. In these circumstances, a typical pipeline will involve removing low quality reads, collapsing of raw reads into unique reads, demultiplexing samples, discarding short reads (typically less than 15 nucleotides) and trimming the adaptors prior to mapping. One of the most commonly used tools is the FASTX_toolkit (http://hannonlab.cshl.edu/fastx_toolkit/), which provides a number of functions to accomplish all of these steps. Other alternatives, with more limited functions include Cutadapt [45], Trimmomatic [46], PRINSEQ [47] as well as custom scripts.
2. Mapping to reference genomes: The reads that pass the above filtering steps are mapped onto reference genomes or transcriptomes. The most commonly used mapping algorithms used for this task include Bowtie [48], Bowtie2 [49], STAR [50], Novoalign (http://www.novocraft.com/products/novoalign/), RMAP [51], TopHat [52], GSnap [53], SOAP [54] and BWA [55], some with unique advantages over others depending on whether mapping is done on a genome versus transcriptome. The choice of algorithm and the parameters for mapping will need to be finely tuned depending on which CLIP methodology is employed and the properties of the RBP of interest. For example, PAR-CLIP reads are expected to contain a number of T-to-C substitutions, and thus mismatches (typically ≤ 2 for reads between 15 and 40 nucleotides) should be allowed during mapping. While all algorithms allow mapping with mismatches, not all can handle deletions, which arise as a result of UV_254_ nm crosslinking in HITS-CLIP and related methods. For example while the original Bowtie algorithm did not allow gaps during alignment, Bowtie2 was developed to enable alignments with indels. Similarly, if mapping is done on transcriptomes, alignment algorithms such as STAR, which allow higher accuracy and speed for mapping spliced transcripts should be preferred. However, mapping to the transcriptome will clearly lead to the exclusion of reads derived from introns, which may constitute the primary binding sites for various splicing regulatory proteins. Thus, a general strategy whereby CLIP reads are mapped first to the transcriptome and the remaining reads are mapped to the genome may work the best for proteins for which there is no information on the types of targeted RNA molecules.
3. Peak calling: The next essential step in CLIP analysis is identification of the true binding sites by what is often referred to as *peak calling*. In simple terms, peak calling is the process by which clusters of reads that map to distinct locations are separated from background reads that may stem from unspecific binding events or contaminants during the CLIP procedure. Peaks are typically defined based on a number of variables such as read depth relative to surrounding regions, presence of expected and absence of unwanted mutations (as in the case of PAR-CLIP-based approaches) and peak shape. While peak calling can be based solely on CLIP data, additional controls such as data derived from replicates and negative controls (i.e. immunoprecipitations done with isotype controls and/or conducted in lysates lacking the RBP of interest) can further increase specificity of peak calling. Comparison of the CLIP peaks with transcript abundance derived from matching RNA-seq experiments allows the discrimination of whether a binding event is merely a result of transcript abundance or a more specific interaction between the RBP and its target RNA. Several peak calling programs have been developed and include Piranha [56], CLIPper [57], PIPE-CLIP [58], Pyicos [59] that work with all CLIP variants, and PARalyzer [60] and wavClusteR that are specifically developed for PAR-CLIP analysis. For more details on the statistical models underlying these programs, we refer the readers to detailed reviews on this topic [41, 42].
4. Post-processing analyses: Following the identification of peaks, further analyses are typically conducted to identify the specific rules that may determine protein binding. For example, many studies generally assess what classes of RNAs and where within those transcripts binding sites are located and whether there are distinct motifs within the binding sites. While the former analyses are done usually by custom scripts, programs such as MEME [61], HOMER [62] and cERMIT [63] are commonly used for motif discovery. Finally, binding sites derived from CLIP experiments can further be analyzed by programs that are commonly used in gene expression profiling experiments for gene ontology and pathway analyses.

## Application of CLIP techniques in retrovirology

### Novel insights into selective HIV-1 genome packaging

All major steps of HIV-1 particle assembly are orchestrated by the major structural protein, Gag [6]. Gag undergoes major changes in its subcellular localization, structure and oligomeric state during this process. Immediately following its synthesis, Gag exists as a diffuse pool of monomers and low-order multimers in the cytosol, where it initially binds to the viral RNA genome [64, 65]. Concurrent with binding to the plasma membrane Gag undergoes a major structural change and oligomerizes around the viral genome [65]. Following the release of immature particles from the host cell’s plasma membrane, particles undergo maturation—Gag is subjected to several proteolytic cleavages, which liberates NC and other constituent domains. NC remains bound to the viral genome and condenses with it inside the remodeled conical capsid lattice. Thus, a crucial property of Gag is its ability to select two copies of the viral genome for packaging in the cytosol and remain bound to them through various subcellular settings and configurations.

The mechanism by which HIV-1 selectively packages a dimeric unspliced viral genome is based largely on prior observations with simple retroviruses, as well as genetic studies and limited in vitro data. Selective packaging of the HIV-1 genome is governed in part by binding of the nucleocapsid (NC) domain of Gag to a highly structured *cis*-acting packaging element, psi (Ψ), within the 5′ leader of the viral genome, composed of sequences in the unique 5′ region (U5) and between the tRNA primer binding site (PBS) and the 5′ portion of the Gag open reading frame (ORF). However, disruption of Ψ only modestly decreases HIV-1 RNA encapsidation [66–68], and sequences outside Ψ can increase virion RNA levels and viral vector titers [69–73]. In addition, viral RNA is not necessary for particle assembly and cellular RNAs can be packaged in its absence [74, 75]. Thus, although several lines of evidence have long indicated that sequences other than Ψ can contribute to genome packaging, determining the identities and features of these elements remained a challenge, due largely to lack of proper assays to study this process in cells.

Application of the CLIP methodology to the study of Gag-RNA interactions during different stages of particle assembly in cells revealed previously unanticipated rules of selective genome packaging [14]. First, nucleotide-resolution mapping of Gag binding to the HIV-1 genome in the cytosol revealed selective binding to sequences that coincide nearly precisely with a minimal element that can drive genome packaging. This minimal psi (Ψ) element adopts alternative structures, one of which favors genome packaging [76–78]. Second, in addition to Ψ, cytoplasmic Gag was bound to additional discrete elements on the viral RNA, including Rev Responsive Element (RRE), another highly structured region that mediates the export of HIV-1 RNAs from the nucleus. Although Gag-RRE interactions appeared to be dispensable for genome packaging, a more recent study has implied a role for it in preventing Gag from moving away from the viral RNA genome in the cytosol [79]. Third, mapping of Gag binding sites within the cellular mRNAs revealed a striking contrast between the binding preference of cytosolic versus membrane-bound Gag; while cytosolic Gag preferentially bound to GU-rich motifs, A-rich mRNA sequences were found to be enriched in plasma membrane-bound mRNA molecules. Remarkably, the nucleotide composition of the cellular mRNA targets of Gag at the plasma membrane mirrored the unusual A-rich nucleotide composition of the HIV-1 genome [14]. Finally, upon proteolytic cleavage of Gag in mature virions, the NC binding preference reverted back to GU-rich mRNA sequences and discrete viral RNA elements including Ψ. Together, these findings suggest that upon binding of monomeric Gag to the viral genome through Ψ, multimerization-dependent changes in the RNA binding specificity of Gag may drive the selective packaging of the A-rich viral genome. In line with this model, a recent study has shown that longer segments of the Gag ORF, but not Ψ alone, can gradually increase the packaging of heterologous RNAs into virions [80]. Thus as part of the selective RNA packaging process, the role of Gag-Ψ interaction may be to nucleate further assembly of Gag oligomers on the viral genome [81].

### HIV-1 MA-tRNA interactions

In addition to the NC domain, the matrix (MA) domain of Gag had long been suspected to bind RNA, based largely on in vitro assays [82–87]. The N-terminal basic amino acids of MA that are thought to bind RNA also mediate binding to cellular membranes [83, 88–92]. However, MA-RNA interaction has been thought to be fairly non-specific, and whether it actually occurs in cells could not be addressed until the application of the CLIP methodology. By releasing MA from Gag by Factor Xa protease-mediated cleavage as part of the CLIP procedure, following UV-crosslinking of Gag-expressing cells, MA was bound to a specific set of tRNAs in the cytosol [36]. In fact, MA-tRNA interactions constituted the most frequent binding event between cytosolic Gag and RNA. Notably, MA-tRNA interaction was lost upon binding of Gag to the plasma membrane and RNase treatment of cell lysates expressing Gag led to significantly higher levels of membrane associated Gag [36]. Together, these findings suggested that occlusion of MA basic residues by specific tRNAs may target HIV-1 assembly to the plasma membrane and prevent nonproductive assembly on intracellular membranes. Alternatively, tRNA binding by MA may temporally regulate membrane binding and assembly [93]. Recent in vitro liposome binding assays also revealed that a specific set of RNAs, including Ψ, total yeast tRNA and tRNA^Pro^ can inhibit Gag binding to negatively charged lipid membranes lacking PI(4, 5)P_2_ [94]. Interestingly, tRNA^Lys^, which was one of the most frequently bound to tRNAs by MA in cells [36], did not prevent Gag binding to liposomes [94]. As this study only tested the ability of in vitro transcribed tRNAs in regulating Gag membrane binding, it remains to be seen whether tRNAs containing the complete set of post-transcriptional modifications exhibit differences in MA binding in vitro.

In addition to regulation of Gag membrane binding, MA-tRNA interactions could have other functions. An obvious possibility is regulation of viral and/or host translation. As a result of the unusually A-rich nature of the HIV-1 genome [95–97], Ile, Lys, Glu and Val codons are overrepresented in the Gag and Pol ORFs [98]. Notably, tRNA^Lys^, tRNA^Glu^ and tRNA^Val^ were found to be amongst the most frequently bound by MA, suggesting the possibility of MA enhancing the translation of Gag and Pol by sequestering these specific set of tRNAs. Alternatively, it is conceivable that by sequestering tRNAs, MA could inhibit translation of host mRNAs whose products may block viral replication. Indeed, one report has suggested that interaction of MA with host translation elongation factors via a tRNA bridge could inhibit in vitro translation [84]. It remains to be determined whether MA-tRNA interactions in a relevant infection setting can influence viral or host translation. Finally, it is possible that if not bound by tRNAs, the basic patch on MA may nonspecifically bind to the viral genome and even prevent the proper interaction of NC with the genome, which may inhibit subsequent steps of infection. In a similar scenario, MA binding to small RNAs might be a mechanism to avoid aggregation by a protein that has two distinct RNA binding domains and an intrinsic tendency to multimerize.

### Role of IN-RNA interactions in particle maturation

The morphological changes that occur during HIV-1 particle maturation are often thought to be dependent only on proteolytic cleavage of Gag. The cleaved CA domain of Gag forms the conical lattice within which the viral genome condenses, along with the cleaved NC domain of Gag as well as viral enzymes integrase (IN) and reverse transcriptase (RT), cleavage products of the Pol polyprotein. However, more than two decades ago, mutational studies of the HIV-1 IN indicated that it may also play an active role in proper particle maturation [99–110]. In particular, a set of mutations referred to as Class II IN mutations, were shown to lead to the formation of morphologically aberrant “eccentric” particles, in which the viral ribonucleoproteins complexes (vRNPs) are mislocalized outside the conical CA lattice [101, 103, 111]. Although IN is known to bind DNA through several charged residues scattered throughout the protein (reviewed in [112]) and can bind to RNA in vitro with some specificity [113], why and how mutations within IN would specifically lead to mislocalization of vRNPs in virions remained enigmatic.

The recent development of allosteric integrase inhibitors (ALLINIs) reignited research in this area. While ALLINIs were initially developed to target IN binding to the cellular cofactor LEDGF, it was later shown that these compounds primarily act during particle maturation and lead to morphological aberrations in particles similar to those induced by the aforementioned Class II IN mutations [114–119]. Biochemical analysis of IN in vitro and in virions revealed that ALLINIs induce aberrant IN multimerization [103, 111, 120–123] through catalytic core domain–C-terminal domain interactions at the dimer–dimer interface [116]. By employing CLIP and complementary in vitro approaches, recent studies have shown that low-order multimers of IN binds to distinct structured elements on the viral genome, including TAR, with high affinity [13]. Notably, while ALLINIs indirectly block these interactions by inducing IN oligomerization, mutations of basic amino acids within the C-terminal domain of IN can abolish IN-RNA binding directly without altering the multimeric state of IN. Inhibition of IN-RNA interactions leads to mislocalization vRNPs and IN outside the conical capsid core [124]. Surprisingly, CLIP experiments reveal that the pattern of NC binding on the vRNA genome seems to be unaffected by IN mutations or ALLINIs, despite the mislocalization of vRNPs in eccentric particles [124]. Together, these aberrations in virion morphology are accompanied by premature degradation of vRNPs and IN, and spatial separation of RT from vRNPs, explaining the early reverse transcription block of these particles in target cells [124]. Thus, CLIP has been key in unveiling the key role of IN-RNA interactions during virion morphogenesis that ensure the correct localization of core components inside the CA lattice during particle maturation.

### Incorporation of APOBEC3 proteins into virions

While viral RNAs contain sequence and structural elements that regulate key steps in HIV-1 replication, they can also be recognized by host defense mechanisms. Infiltration of the host APOBEC3 (A3) proteins into virus particles by binding viral RNAs is a prime example of this process. A3 proteins are a family of cytidine deaminases that inhibit the replication of a broad range of viruses and retroelements (reviewed in [125, 126]). A3s inhibit replication in two ways. One mechanism involves the deamination of cytidines to uridines in (–) strand DNA during reverse transcription, resulting in the accumulation dG-to-dA mutations on the coding strand [127–130] and lethal hypermutation. Additionally, A3 proteins have been shown to induce a deamination-independent block, by binding to reverse transcriptase and inhibiting reverse transcription [131–135]. Packaging of A3 proteins into HIV-1 virions is required for their antiviral activity and depends on the NC domain of Gag and its associated RNA [136–141]. A3 proteins appear to be promiscuous RNA binding proteins and it has been difficult to determine whether they selectively target viral or cellular RNAs to infiltrate into particles. For example, there is evidence to indicate that viral genome [142], 7SL RNA, a cellular RNA that is normally part of the signal recognition particle and is enriched in retroviral particles [143], or both cellular and viral RNAs [140, 141] can mediate packaging of A3G into particles. As many of these studies largely relied only on genetic assays, whether A3 proteins exhibit any preference towards a specific set of RNAs, or sequence features within them in a relevant setting remained unknown. Nevertheless, the presence of a discrete RNA binding domain in A3G implies some level of selectivity in RNA binding, much like other RBPs [144, 145].

Three recent studies employing CLIP have provided insight into the RNA-binding properties of several A3 proteins in infected cells and in virions [146, 147]. The earlier iCLIP-based study indicated that although the viral genome is enriched amongst A3F and A3G-bound RNAs, a diverse set of RNAs could drive the incorporation of A3F and A3G into virions [146]. A subsequent PAR-CLIP-based study confirmed some of these findings in that A3 proteins were shown to bind similar classes of cellular RNAs and HIV-1 RNA was bound preferentially over cellular RNAs in infected cells. However, the PAR-CLIP approach provided a higher resolution assessment of A3-RNA interactions in cells, likely due to the ability to more accurately identify the site of crosslinking. Most importantly, detailed analysis of A3 binding sites revealed that the A3 proteins partly mimic the RNA-binding specificity of NC, in that they target RNA sequences that are G-rich and A-rich [147]. This model provides some explanation of how A3 proteins are incorporated efficiently into virions in the presence of a vast excess of cellular RNA molecules. This model invokes a bias in the binding of A3 proteins to RNA molecules of a given sequence composition, as a way of maintaining broad RNA binding specificity, while removing the need to occupy all mRNA sequences present in an infected cell. One recent study, the first to reveal a crystal structure of an A3 protein in complex with an RNA showed that the A3H protein has a particular propensity to bind to seven-nucleotide duplexes, in a manner that was independent of the nucleotide sequences forming the duplexes [148]. Accompanying CLIP experiments showed that the sites in the HIV-1 genome to which A3H was most frequently bound were invariably predicted to contain 7nt duplexes.

### Role of zinc finger antiviral protein (ZAP) in imposing compositional bias on viral genomes

The genomes of vertebrates are marked with a paucity of CG dinucleotides [149], a feature that is well understood to have been caused by the action of CG-specific DNA methyl transferases and methyl-cytosine deamination, over hundreds of millions of years. More mysteriously, inspection of the composition of the genomes of RNA viruses in vertebrates, reveal that they mimic this CG-poor state, even though they are not substrates for DNA methyl transferases [150–152]. Recent work, in the context of HIV-1 has shown that the paucity of CG dinucleotides is essential for viral replication, and that the appearance of too many CG dinucleotides in the viral genome causes cytoplasmic depletion of viral RNA [153]. The apparently destabilizing effect of CG dinucleotides was cumulative, and found to be induced by CG dinucleotides in both translated portions of an mRNA and also in untranslated exons. Further experiments showed that zinc finger antiviral protein (ZAP) [154] a protein that encodes four CCCH zinc fingers in is N-terminal domain is essential the for mediating the deleterious effects of CG dinucleotides. Indeed, HIV-1 mutants containing segments whose CG-content mimicked a random nucleotide sequence could not replicate in unmanipulated cells containing an intact *ZAP* gene, but could replicate with wild-type kinetics in cells rendered ZAP-deficient by CRISPR-Cas9 editing [153].

While previous studies had shown that ZAP had antiviral activity against a number of RNA viruses, several conventional techniques could not identify a common sequence motif or RNA feature that could explain how ZAP was able to specifically target viral RNA sequences [154, 155]. RNA elements that could confer sensitivity to ZAP when inserted into a reporter RNA were large, leading to the proposal that a specific tertiary structure constituted a ZAP recognition site. However, RNA elements that conferred sensitivity to ZAP did so in both orientations [156], effectively refuting these models. CLIP experiments showed unambiguously that ZAP binds directly and selectively to RNA elements that contain CG dinucleotides, but exhibits no preferential binding to RNA elements containing GC or any other dinucleotide [153]. Interestingly, these results suggest that ZAP arose to exploit a compositional difference between host mRNAs and RNAs from viruses have high CG content. However, the dinucleotide composition of HIV-1, appears to have adapted to evade ZAP and it is possible that ZAP has driven the purging of CG dinucleotides from a range of RNA viruses.

### Identification of m^6^A marks on HIV-1 RNAs

Like proteins and DNA, RNA can undergo a number of chemical modifications that subsequently affect its metabolism, function and localization. While tRNAs and rRNAs are subjected to the most diverse set of modifications, recent transcriptome-wide studies revealed the presence of numerous mRNAs modifications [157–162]. Methylation of adenosine at the N6 position (m^6^A) is the most prevalent of these and has been proposed to regulate several aspects of RNA metabolism, including splicing, nuclear export, localization, stability and translation [163]. m^6^A modification is catalyzed by a nuclear “writer” protein complex, composed of two methyltransferase-like enzymes, METTL3 and MTTL4, and their cofactor Wilms tumor 1-associated protein (WTAP). This modification can be reversed by two RNA demethylases, or ‘‘erasers’’, ALKBH5 (a-ketoglutamarate-dependent dioxygenase homolog 5) and FTO (fat mass and obesity associated). m^6^A-modifications on mRNAs can be bound by three related cytosolic ‘‘reader’’ proteins called YTH-domain containing family 1 (YTHDF1), YTHDF2, and YTHDF3. Exactly how binding of these proteins on modified nucleotides regulate mRNA metabolism is currently unknown. Nonetheless, m^6^A modifications can be found on mRNAs of diverse viruses that replicate in the nucleus, including SV40 [164], adenovirus [165, 166], influenza A virus [167] as well as retroviruses such as avian sarcoma virus [168] and Rous sarcoma virus [169, 170]. Until recently, whether HIV-1 mRNAs contained m^6^A modifications and how this affected virus replication was not known.

Three recent studies have addressed this question by immunoprecipitating methylated HIV-1 RNAs from infected cells using a m^6^A-specific antibody followed by high throughput sequencing of the immunoprecipitated mRNAs [171–173]. Strikingly, there was virtually no overlap in the m^6^A sites identified in these independent studies. This lack of consistency can in part be explained by the different approaches taken. The first published study that has utilized a RIP-seq approach, in which m^6^A-modified RNAs were immunoprecipitated from cell lysates and sequenced, found m^6^A modifications throughout the viral genome [172]. In contrast, a later study, which included a PAR-CLIP-based crosslinking step following immunoprecipitation of m^6^A-modified RNAs, found that the m^6^A modifications were exclusively localized within the viral 3′ UTRs [171]. Importantly, parallel YTHDF PAR-CLIP experiments conducted in this latter study revealed binding sites at or near the modified nucleotides, reinforcing the findings from m^6^A-specific immunoprecipitations [171]. A third study similarly coupled YTHDF HITS-CLIP with m^6^A-seq [173] and identified putative modification sites within 3′ and 5′ UTRs of HIV-1 mRNAs. Notably, none of these sites overlapped with those identified in the former studies. Thus, while CLIP methodologies have been highly instrumental in identification of m^6^A sites on HIV-1 RNAs, cross-validation of reagents (i.e. cell lines, viruses, m^6^A antibodies) and methods (i.e. m^6^A-seq, PAR- vs. HITS-CLIP) will be necessary to reach to a consensus in future studies.

## Conclusions

Application of the CLIP methods to questions in retrovirology will undoubtedly continue to increase, given the large number of RBPs that are known and continuing to emerge as key regulators of retroviral replication. Several poorly explored areas in retrovirology will benefit from these approaches. One of the immediate applications of this methodology will be in determining how the alternative splicing of HIV-1 transcripts is regulated by cellular hnRNP and SR splicing-regulatory proteins. Although the families of hnRNP and SR proteins constitute more than 50 proteins, only a few have been shown to play roles in HIV-1 RNA splicing. In addition, none of the studies performed to date determined where on viral RNAs these proteins bind. Instead, in vitro splicing reporters and genetic assays were used, which are prone to artefacts. Another exciting area of research where CLIP and related methodologies may make a major impact is the sensing of viral nucleic acids in infected cells. HIV-1 infection induces high levels of interferon and other cytokines during the acute phase of infection, suggesting that viral nucleic acids are sensed in infected cells. While a few isolated studies indicated that viral reverse transcription products or RNA elements can be sensed in certain settings, it remains to be determined what features of viral nucleic acids are sensed and whether viral RNA or DNA elicits an inflammatory response. While A3 proteins provide a good example of how viral RNAs can be targeted by antiviral host proteins, it is plausible that many other cellular proteins that can recognize and target viral RNAs. CLIP will be a key tool in unveiling novel cellular proteins that participate at the HIV-1-host interface. Finally, although CLIP has so far only been applied to HIV-1 biology, it will certainly find broad applications in retrovirology and virology more generally as the methods and next-generation sequencing becomes more accessible.
